# Impact of socioeconomic status on mortality and morbidity in patients with severe sepsis and septic shock

**DOI:** 10.1186/1757-7241-23-S1-A21

**Published:** 2015-07-16

**Authors:** Amal Schnegelsberg, Julie Mackenhauer, Marie Jessen, Helle L Nibro, Pia Dreyer, Hans Kirkegaard

**Affiliations:** 1 Research Center for Emergency Medicine, Aarhus University Hospital, Aarhus, Odense, Denmark; 2 Department of Anaesthesiology, Regional Hospital of Randers, Randers, Denmark; 3 Intensive Care Unit, Aarhus University Hospital, Aarhus, Denmark; 4 Department of Anaesthesiology and Intensive Care Medicine, Aarhus University Hospital, Skejby, Denmark

## Background

Previous studies on sepsis have focused on early identification and mortality, while studies examining the association between socioeconomic status (SES) and mortality and morbidity in septic patients are sparse.

## Methods

We conducted a historical cohort study at the Intensive Care Unit (ICU), Aarhus University Hospital. All adult patients admitted to the ICU with severe sepsis or septic shock from 2008-2010 were included. Transfers from other ICUs and patients with limited interventions (DNI/DNR) were excluded. Data on patient SES (educational level and personal income), Charlson Comorbidity Index (CCI), readmissions, and mortality were obtained from public registries. Through Kaplan-Meier curves and Cox proportional hazards models, we examined the effect of SES on in-hospital mortality, 1-year all-cause mortality, and time to first acute readmission within one year.

## Results

388 patients were included. Median age was 64 years IQR [55-74], 53% were men. 47% were admitted directly from the emergency department (ED). Median CCI was 2 IQR [1-3], median SAPS II was 40 IQR [30-53]. 52% had septic shock within 24 hours of admission. 1-year mortality in the entire cohort was 46%, of these 61% died in-hospital. Kaplan-Meier curves for in-hospital mortality (n = 388) showed a significant association to income category with a hazard ratio (HR) of 1.59 (95% CI [1.0; 2.5]). Curves for 1-year mortality (n = 278) showed a tendency of worse outcome among the lowest SES groups with the strongest trend in income categories. After adjusting for demographic characteristics, CCI, LOS and SAPS II, the low income group had a significantly increased HR of 1.88 (95% CI [1.0; 3.4]) compared to high income group for 1-year mortality.

56% of hospital survivors experienced a readmission within one year from discharge. Kaplan-Meier curves showed a consistent trend towards reduced time to readmission in the low compared to the higher groups for both income and education.

## Conclusion

Our data suggest that low income affects in-hospital mortality. After discharge, patients in the low income category and with low educational level have a tendency towards higher 1-year mortality as well as reduced time to readmission.

